# Effects of Resistance-Based Multicomponent Training Incorporating Respiratory Exercise on Diaphragm Thickness, Hepatic Attenuation, and Quality of Life in Healthy Older Men: A Randomized Controlled Trial

**DOI:** 10.3390/healthcare14213801

**Published:** 2026-11-06

**Authors:** Coşkun Yılmaz, Hakan Hüseyin Soylu, Okan Demir, Mehmet Bayansalduz

**Affiliations:** 1 Faculty of Sport Sciences, Gümüşhane University, Gümüşhane 29000, Türkiye; 2 Gümüşhane Provincial Health Directorate, Radiology Department, Kelkit District State Hospital, Gümüşhane 29600, Türkiye; 3 Şiran Mustafa Beyaz Vocational School, Gümüşhane University, Gümüşhane 29700, Türkiye; demir@gumushane.edu.tr; 4 Faculty of Sport Sciences, Topkapı University, İstanbul 34087, Türkiye

**Keywords:** resistance-based exercise, healthy aging, older men, diaphragm morphology, respiratory muscle, health-related quality of life, exercise intervention, randomized controlled trial

## Abstract

**Background:** Aging is accompanied by progressive alterations in musculoskeletal and respiratory systems that may contribute to declines in physical capacity and overall health. Although resistance exercise is widely recognized as an effective non-pharmacological strategy for healthy aging, its potential to induce measurable adaptations in respiratory muscle morphology remains insufficiently understood. This study aimed to investigate the effects of a short-term resistance-based multicomponent training program incorporating respiratory exercises on diaphragm thickness, hepatic attenuation, functional capacity, and health-related quality of life in healthy older men. **Methods:** Thirty community-dwelling healthy men aged 60–75 years were randomly allocated to a resistance-based exercise group (RT, n = 15) or a control group (CG, n = 15). The RT group participated in a 4-week resistance-based multicomponent training program incorporating structured respiratory exercises. CG maintained their usual lifestyle. Diaphragm thickness was assessed as the predefined primary outcome using non-contrast computed tomography. Functional walking capacity was assessed using the six-minute walk test (6MWT), while health-related quality of life was evaluated using the Physical Health (PH) and Mental Health (MH) components of the SF-12 questionnaire. Hepatic attenuation was assessed as an exploratory imaging outcome. Group × time interactions were examined using two-way repeated-measures analysis of variance. **Results:** The RT group demonstrated a significant increase in diaphragm thickness compared with the CG (group × time interaction: F = 21.058, *p* < 0.001, *η*^2^p = 0.429). Functional walking capacity also improved substantially in the RT group, with 6MWD increasing from 568.80 ± 42.87 m to 615.53 ± 52.06 m (+46.73 m; +8.22%), whereas the CG showed a slight decline; the group × time interaction was significant (F = 66.604, *p* < 0.001, *η*^2^p = 0.704). No significant group × time interactions were observed for SF-12 Physical Health or Mental Health, whereas hepatic attenuation showed an exploratory group × time effect. **Conclusions:** Four weeks of resistance-based exercise was associated with a significant differential increase in diaphragm thickness and a substantial improvement in functional walking capacity in healthy older men. These findings suggest that short-term exercise may induce measurable morphological and functional adaptations during aging, although the functional significance of the observed diaphragm change remains uncertain. No intervention-specific effects were detected for health-related quality of life, while the hepatic attenuation finding should be considered exploratory. Longer and adequately powered trials incorporating validated respiratory, functional, and metabolic outcomes are warranted.

## 1. Introduction

Population aging is accompanied by progressive changes in musculoskeletal, respiratory, and metabolic health, which may contribute to declines in physical function, exercise capacity, and independence. As life expectancy continues to increase, preserving health span and functional capacity has become an important objective of preventive healthcare and healthy-aging strategies. Regular physical activity and structured exercise are among the most accessible non-pharmacological approaches for maintaining physical function and reducing the consequences of age-related physiological decline [1,2]. In particular, resistance-based exercise is widely recommended for older men because of its established effects on muscular strength, physical performance, functional capacity, and the prevention of age-related functional deterioration [2,3,4,5]. These effects are particularly relevant to healthcare-oriented approaches to aging because maintaining physical capacity may contribute to prolonged functional independence and reduced vulnerability to disability.

Although the musculoskeletal benefits of resistance exercise during aging have been extensively investigated, considerably less is known about whether structured resistance-based exercise is also associated with measurable adaptations in the respiratory musculature. The diaphragm is the principal muscle of inspiration and plays an essential role in ventilation and respiratory mechanics. Aging may be accompanied by alterations in respiratory muscle structure and performance, potentially reducing ventilatory reserve and contributing to limitations in exercise tolerance [2,3,4]. Previous studies have demonstrated that respiratory muscles remain responsive to targeted exercise interventions and that diaphragm morphology can adapt to changes in respiratory loading [2,3,4,5,6,7,8,9]. However, much of the existing evidence has focused on inspiratory muscle training or clinical populations, whereas the potential effects of a broader structured resistance-based exercise intervention on diaphragm morphology in healthy older men remain less well characterized. This gap is relevant to healthy aging because preservation of respiratory muscle integrity may contribute to maintaining exercise tolerance and physical capacity during later life.

Beyond morphological adaptations, preservation of functional capacity is a central component of healthy aging. Functional walking capacity provides an accessible indicator of mobility and physical performance in older men and can be evaluated using the six-minute walk test (6MWT). The six-minute walk distance (6MWD) integrates multiple physiological and functional components required for sustained walking and has been widely used to evaluate exercise-related changes in functional performance in aging and clinical populations. Previous evidence suggests that resistance-based exercise can improve 6MWD in some older populations, although the magnitude and consistency of these responses vary according to participant characteristics, baseline functional status, intervention duration, and exercise modality [9,10]. Thus, assessment of functional walking capacity alongside diaphragm morphology may provide a more comprehensive perspective on the potential effects of short-term exercise in older adults. Importantly, an improvement in 6MWD should be considered a functional outcome in its own right and should not be assumed to result directly from changes in diaphragm morphology in the absence of direct mechanistic or associative analyses.

In addition to objective functional outcomes, health-related quality of life represents an important patient-centered dimension of healthy aging. Regular exercise has been associated with improvements in perceived physical and psychological health in older adults, although the magnitude of these effects may depend on intervention duration, baseline health status, exercise modality, and participant characteristics [11,12,13,14]. Accordingly, examining health-related quality of life together with objective functional and morphological outcomes may help determine whether short-term physiological adaptations are accompanied by broader changes in perceived health. Exploratory assessment of hepatic attenuation may also provide preliminary information regarding potential metabolic adaptations to exercise; however, imaging-derived hepatic attenuation should be interpreted cautiously because non-contrast single-region CT measurements do not provide a direct quantitative assessment of hepatic attenuation [3,15].

Despite increasing evidence supporting exercise as a strategy for healthy aging, an important knowledge gap remains regarding whether a relatively short structured exercise intervention can simultaneously influence respiratory muscle morphology and functional walking capacity in otherwise healthy older men. In particular, few randomized controlled studies have examined diaphragm morphology together with an objective measure of functional walking performance and patient-reported health outcomes within the same intervention framework. Addressing this gap may help clarify whether exercise-related adaptations extend beyond the traditionally studied musculoskeletal outcomes and may contribute to a more multidimensional understanding of exercise-based healthy aging.

Therefore, the primary aim of this randomized controlled trial was to determine whether a four-week structured resistance-based exercise intervention was associated with measurable changes in diaphragm thickness in healthy older men. Secondary outcomes included functional walking capacity assessed by the 6MWT and health-related quality of life assessed using the Physical Health (PH) and Mental Health (MH) components of the SF-12 questionnaire. Hepatic attenuation was examined as an exploratory imaging outcome. We hypothesized that the exercise intervention would be associated with an increase in diaphragm thickness and an improvement in functional walking capacity, whereas changes in patient-reported health and hepatic attenuation were considered exploratory and requiring confirmation in larger and longer-term studies.

## 2. Materials and Methods

### 2.1. Research Design

This study was designed as a parallel-group, two-arm, randomised controlled trial (RCT) with a pre-test and post-test design, conducted in accordance with the CONSORT 2025 guidelines [16]. Ethical approval was granted by the Scientific Research Ethics Committee of Gümüşhane University (Date: 14 June 2023; Decision No: E-95674917-108.99-181733). Prior to enrolment, all participants were extensively briefed on the study procedures and provided written informed consent in adherence to the Declaration of Helsinki. Participants were explicitly informed, both verbally and in writing, of their right to withdraw from the study at any point without penalty or impact on their routine medical care. The study was prospectively registered in ClinicalTrials.gov (NCT07449767) on 27 February 2026, before enrolment of the first participant. The first participant was enrolled on 10 March 2026, and the intervention commenced on 15 March 2026. Primary outcome data collection was completed on 25 April 2026, and the remaining secondary outcome assessments and adverse-event monitoring were completed on 30 May 2026. The study was therefore completed on 30 May 2026.

#### 2.1.1. Participants and Randomization

The study aimed to evaluate the effects of a resistance-based multicomponent training (RT) intervention on diaphragmatic thickness, exploratory hepatic attenuation marker, and quality of life (SF-12) in elderly men (aged 60–75 years). Initially, 38 older men were screened for eligibility. Eight individuals were excluded due to refusal to participate (n = 2), scheduling conflicts (n = 2), and health-related contraindications (n = 4). The remaining 30 participants were randomly assigned in a 1:1 ratio to either the resistance-based multicomponent training group (RT, n = 15) or the Control Group (CG, n = 15). Randomization was performed using a computer-generated sequence (randomizer.org) by an independent researcher blinded to the assessment and intervention phases. Allocation concealment was strictly maintained using sequentially numbered, sealed, opaque envelopes, which were opened only after the completion of baseline measurements.

#### 2.1.2. Blinding and Supervision

Because the exercise intervention could not be masked from participants, participants were not blinded to group allocation. However, the radiologist performing the CT-based diaphragm thickness assessments and the statistician conducting the analyses were blinded to group allocation. Thus, the study was open-label with respect to participants, while outcome assessment and statistical analysis were performed under blinded conditions. The RT group underwent a structured resistance-based multicomponent training programme, whereas the CG were instructed to maintain their habitual daily routines. For participant safety, all intervention stages were supervised by a physician.

### 2.2. Participants and Sample Size Calculation

The baseline demographic and anthropometric characteristics of the experimental (RT; n = 15) and control (CG; n = 15) groups are presented in Table 1.

An a priori sample size calculation was performed using G*Power version 3.1.9.7 (Heinrich Heine University Düsseldorf, Düsseldorf, Germany) [17]. The analysis was conducted using an F test for ANOVA with repeated measures, within-between interaction, corresponding to the planned Group × Time interaction for the primary outcome, diaphragm thickness. The assumed effect size was Cohen’s f = 0.40, corresponding approximately to a partial eta squared of *η*^2^p= 0.14, based on the relationship f = √ [*η*^2^p/(1 − *η*^2^p)]. The effect size was selected a priori according to Cohen’s conventional criterion for a large effect size [3]. The analysis was performed with an alpha level of 0.05 and statistical power of 0.95, with two groups and two repeated measurements. The assumed correlation between repeated measures was 0.50, and the nonsphericity correction was set to ε = 1.0. Under these assumptions, the minimum required total sample size was 28 participants (14 per group). To minimise the potential risks to health and the risk of having to stop working, each group consisted of 15 people.

#### Eligibility Criteria

Inclusion criteria were: (1) aged 60–75 years, (2) no musculoskeletal injuries within the last six months, (3) normal cognitive function and neuropsychological stability, (4) no participation in any rehabilitation program within the past 12 months, and (5) the ability to perform activities of daily living (ADL) independently.

Exclusion criteria included: (1) uncontrolled cardiovascular or pulmonary diseases, (2) chronic liver diseases, (3) recent respiratory tract infections, and (4) use of medications affecting muscle or liver metabolism (e.g., systemic corticosteroids, anabolic agents, hepatotoxic drugs, or lipid-lowering therapies). These rigorous criteria were implemented to minimize the influence of potential confounding factors such as malnutrition, systemic illness, or metabolic disorders on the study outcomes. Prior to enrollment, all participants received verbal and written information about the study and provided written informed consent before participation.

### 2.3. Study Procedure

**Figure 1:**
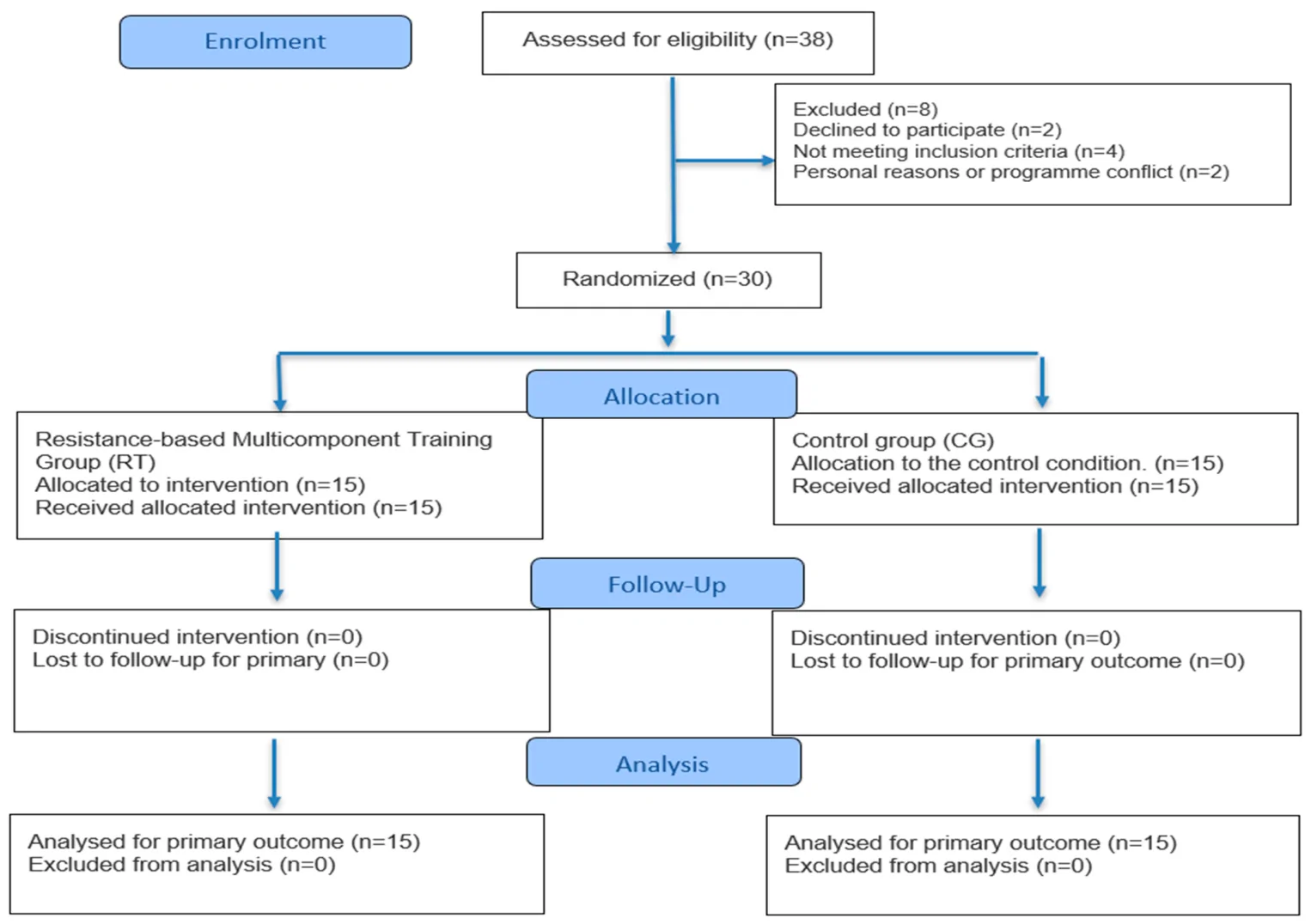
CONSORT flow diagram of participant recruitment, allocation, follow-up, and analysis.

Male participants aged 60–75 years attended three scheduled study visits. During the initial visit, participants received detailed information about the study procedures and underwent an orientation session for the exercise intervention. One week later, the second visit was conducted for baseline assessments before initiation of the intervention. At this study visit, the SF-12 questionnaire was administered, and a standardized non-contrast computed tomography (CT) examination was performed as part of the study protocol to assess both diaphragm thickness and hepatic attenuation. Thus, the baseline CT images were acquired prospectively for the purposes of the present study and were not obtained retrospectively from previously performed clinical examinations. Following completion of the four-week resistance-based exercise intervention, participants attended the third study visit for post-intervention assessments. A second standardized non-contrast CT examination was performed at the prespecified post-intervention assessment time point using the same CT system and acquisition protocol as at baseline. Diaphragm thickness and hepatic attenuation were assessed from the study-specific CT examinations at both time points. All CT examinations were performed according to the standardised imaging protocol described below (Figure 1).

This trial was registered at ClinicalTrials.gov under the title “Effects of Resistance-Based Multicomponent Training Incorporating Respiratory Exercise on Diaphragm Thickness, Hepatic Attenuation, and Quality of Life in Healthy Older Men: A Randomized Controlled Trial’’. ClinicalTrials.gov: NCT07449767, Date: 27 February 2026. (Prospectively).

### 2.4. Outcome Measures

#### 2.4.1. Assessment of Diaphragm Thickness (DT)

Diaphragm thickness was assessed prospectively using non-contrast computed tomography (CT) at two prespecified study time points: baseline, before initiation of the four-week resistance-based exercise intervention, and post-intervention, following completion of the intervention. CT examinations were performed specifically as part of the present study rather than using previously acquired clinical CT scans.

All CT examinations were performed using the same CT system (GE Revolution EVO Cardiac; GE Healthcare, Waukesha, WI, USA) and according to a standardized acquisition protocol. Participants were positioned supine, and image acquisition was performed during an instructed end-inspiratory breath-hold. The acquisition parameters were a tube voltage of 120 kVp, an exposure of 150 mAs with automatic tube current modulation, a pitch factor of 0.98, and a gantry rotation time of 0.5 s. Images were reconstructed with a slice thickness of 1.25 mm using the adaptive statistical iterative reconstruction (ASIR-V 40%) algorithm with a standard soft-tissue reconstruction kernel.

Diaphragm thickness was measured on non-contrast axial CT images at the level of the superior pole of the right kidney. In order to minimise measurement variability, the same anatomical reference point and a standardised imaging and reconstruction protocol were used at both assessment time points [18,19]. Three measurements were obtained for each participant at each time point, and the mean of the three measurements was used for statistical analysis. All CT measurements were performed by an experienced radiologist who was blinded to group allocation. Intra-observer reliability was assessed using the intraclass correlation coefficient (ICC), which was 0.823.

#### 2.4.2. Anthropometric and Body Composition Measurements

Stature and body mass were determined using standardized anthropometric instruments (Seca 769 stadiometer, Seca GmbH, Hamburg, Germany, and Beurer GS27 digital scale, Beurer GmbH, Ulm, Germany). Stature was measured to the nearest 0.1 cm, and body mass was recorded with 0.1 kg precision. All measurements were conducted under standardized conditions, with participants wearing light clothing and being barefoot. Body Mass Index (BMI) was calculated as body mass divided by the square of the stature (kg/m^2^). Although detailed body composition analysis was not performed, BMI values were utilized as a general proxy to assess the baseline body mass status of the participants.

#### 2.4.3. Assessment of Hepatic Attenuation (HA)

Hepatic attenuation was assessed using the same study-specific, prospectively acquired, non-contrast CT examinations performed for the primary assessment of diaphragm thickness. Thus, no additional CT acquisition was performed specifically for hepatic attenuation, avoiding any additional radiation exposure for this secondary/exploratory measurement. CT examinations were performed at baseline, before initiation of the 4-week intervention, and immediately after completion of the intervention using the same CT system (GE Revolution EVO Cardiac; GE Healthcare, Waukesha, WI, USA) and the same standardized acquisition protocol.

To minimize radiation exposure in accordance with the As Low As Reasonably Achievable (ALARA) principle, a single non-contrast CT acquisition was performed at each study time point to obtain both diaphragm thickness and hepatic attenuation measurements [17,18,19]. No intravenous contrast agent, delayed phase, or additional acquisition was performed. The examinations were acquired at 120 kVp using automatic tube current modulation, with a nominal reference tube current of 150 mAs, a pitch of 0.98, a gantry rotation time of 0.5 s, and a reconstructed slice thickness of 1.25 mm using ASiR-V 40% iterative reconstruction and a standard soft-tissue reconstruction kernel. Participants were examined in the supine position and instructed to perform an end-inspiratory breath-hold during image acquisition.

Radiation dose indices were systematically recorded from the scanner-generated DICOM structured dose reports for each examination. The volume CT dose index (CTDI_vol_) and dose-length product (DLP) were recorded separately for the baseline and post-intervention examinations. The CTDI_vol_ ranged from 6.0 to 11.0 mGy for the baseline examinations and from 6.0 to 11.0 mGy for the post-intervention examinations. The corresponding DLP values ranged from 240 to 550 mGy·cm at both time points. Thus, the estimated cumulative DLP across the two study-specific CT examinations was approximately 480–1100 mGy·cm per participant.

The estimated effective dose (ED) was calculated from the DLP using a published adult abdominal CT DLP-to-effective-dose conversion coefficient of 0.015 mSv/(mGy·cm). Accordingly, the estimated ED was approximately 3.6–8.3 mSv per CT examination, and the estimated cumulative ED attributable to the two study-specific CT examinations was approximately 7.2–16.5 mSv per participant. These values represent DLP-derived estimates of effective dose for radiation-protection and protocol-comparison purposes and should not be interpreted as patient-specific absorbed or organ doses.

For hepatic attenuation analysis, a region of interest (ROI) was placed in Segment VIII of the liver adjacent to the middle hepatic vein, avoiding visible vessels, focal lesions, and image artifacts. Three measurements were obtained and averaged to determine the hepatic attenuation value for each examination. Hepatic attenuation infiltration was estimated based on tissue attenuation, with values below 33 Hounsfield Units (HU) serving as a proxy for higher fat content. Consequently, all hepatic density analyses were treated as exploratory [3,18,19]. The same experienced radiologist, blinded to group allocation, performed the measurements using the same procedure at both time points. The intraobserver reliability for hepatic attenuation measurements was high (ICC = 0.821).

Importantly, all CT examinations used in this study were performed prospectively and specifically as predefined components of the research protocol rather than being retrospectively obtained from examinations performed for clinical indications. Only two CT examinations were performed for each participant, one at baseline and one immediately after the 4-week intervention. The use of two examinations was justified by the predefined primary outcome of diaphragm thickness, which required assessment at both baseline and post-intervention time points. Importantly, the same single CT acquisition at each time point simultaneously provided the exploratory hepatic attenuation measurement, thereby avoiding any additional radiation exposure for this secondary outcome.

The potential radiation exposure and associated risks of ionizing radiation were disclosed to participants as part of the informed-consent process, in accordance with the participant information and consent documents approved by the Research Ethics Board. Written informed consent was obtained before study-specific procedures were initiated. Hepatic attenuation was considered an exploratory imaging outcome and was not intended to provide a clinical diagnosis of hepatic steatosis or to serve as an equivalent measure of MRI-derived proton density fat fraction (MRI-PDFF) or controlled attenuation parameter (CAP).

#### 2.4.4. Rate of Perceived Exertion (RPE)

Perceived exertion during exercise was monitored using the Borg 6–20 Rating of Perceived Exertion (RPE) scale. This scale allows for the subjective assessment of an individual’s physical effort during activity [20]. In the present study, RPE was utilized exclusively to monitor and standardize exercise intensity; the recorded scores were not included in the formal statistical analysis.

#### 2.4.5. Six-Minute Walk Test (6MWT) Protocol

Functional exercise capacity was assessed using the 6-Minute Walk Test (6MWT) according to a standardised protocol based on established 6MWT recommendations [21,22]. The test was performed on a flat, straight 30 m indoor corridor, with the course clearly marked at both ends. Participants were instructed to walk back and forth along the corridor for 6 min at a self-selected walking pace, with the objective of covering the greatest possible distance within the allotted time. Participants turned around at the marked endpoints and continued walking without running. Participants wore comfortable footwear suitable for walking and were instructed to use the same footwear at the pre- and post-intervention assessments whenever possible. During the test, the evaluator recorded the time and provided standard encouragement at each minute interval. Standardised verbal encouragement was provided at predetermined intervals using the same wording for all participants. No additional performance-related feedback was provided during the test. The same assessor conducted the 6MWT at baseline and post-intervention whenever possible, using the same testing area, course length, equipment, and procedural instructions. The primary outcome was the total distance walked during the 6 min period, recorded in metres. The evaluator also counted the number of laps completed by each participant. At the sixth minute, participants stopped at their current location on the path, and the evaluator measured the total distance walked for the final lap. The evaluator then calculated and recorded the total distance walked during the 6 min period [21,22]. A familiarisation trial was performed before the baseline assessment. Participants completed a practice 6MWT session prior to the baseline measurement; this was followed by a standard one-week recovery period.

#### 2.4.6. Quality of Life Scale (QoL)

Short Form-36 (SF-36) was developed by Ware et al. (1995) to measure quality of life. SF-12 Quality of Life Scale was formed by taking 12 different items from 8 subscales of SF-36 [23]. A Turkish validity and reliability study was conducted by Soylu and Kütük (2021) [24]. In the scale, the functional status, well-being, and general health perception of the individual are assessed. Questions questioning physical and emotional status are answered as yes or no, and other questions include Likert-type options ranging from 3 to 6. Mental component summary (MH) score is obtained from the mental health, emotional role, social functioning, and energy subcategories, and the physical component summary (PH) score is obtained from the physical role, physical functioning, general health, and body pain subcategories. Scoring ranges from 0 to 100. A higher score is an indicator of better health. The calculation of sub-dimensions in the obtained data was made on https://orthopowertools.com/SF12, accessed on 24 August 2026. The Cronbach’s alpha coefficient of the SF-12 Short Health Scale was calculated as *α*: 0.73 in the physical subscale and *α*: 0.72 in the mental subscale [24]. In our study, it was calculated as *α*: 0.762 in the physical sub-dimension and *α*: 0.732 in the mental sub-dimension.

### 2.5. Intervention

In accordance with the guidelines of the National Strength and Conditioning Association (NSCA), a four-week programme incorporating both conventional resistance exercises and structured breathing components was implemented to support physiological adaptations in older men. Participants completed three supervised sessions per week, with each session lasting approximately 30–40 min. The resistance component included the prescribed strengthening exercises performed at a moderate perceived exertion level (RPE 12–15). Resistance was increased when participants consistently reported an RPE below the prescribed range while completing all repetitions with correct technique. Exercise sets were not prescribed to volitional failure, and proximity to failure was not quantified. In addition, the programme incorporated respiratory exercises throughout the intervention, including diaphragmatic breathing, pursed-lip breathing, balloon-based breathing exercises, and other prescribed breathing activities. Thus, the intervention was designed as a multicomponent training programme rather than as resistance exercise alone (Table 2). No adverse events were reported during the four-week intervention period. No exercise-related injury, serious adverse event, or adverse event requiring medical evaluation or discontinuation of the intervention occurred. All participants completed the intervention and post-intervention assessments. The exercise sessions were delivered by an experienced strength and conditioning specialist, with a physician available for medical safety oversight. In the study, the same training programme was administered to all participants in the RT group in order to rule out the contralateral effect. Each training session was preceded by a standardized 10 min dynamic warm-up designed to enhance joint mobility, elevate core body temperature, and prime the cardiovascular and musculoskeletal systems for the intervention. The routine commenced with 4 min of low-intensity walking to gradually increase heart rate. This was followed by specific dynamic mobility exercises, including 10 repetitions of forward and backward shoulder rotations and controlled cervical movements (flexion, extension, and lateral flexion). Upper body circulation was further stimulated through 2 min of rhythmic aerobic-style arm movements, characterized by controlled forward and lateral swings. Subsequently, 2 min of marching in place with light knee lifts were performed to activate the lower extremity musculature. The warm-up concluded with a 2 min sequence of alternating heel-to-glute movements (butt kicks) to activate the hamstrings. All exercises were executed at a light-to-moderate intensity in a slow, controlled, and pain-free manner. To ensure participant safety and stability, individuals were encouraged to use a chair or wall for support as needed. The respiratory component consisted of structured diaphragmatic breathing, pursed-lip breathing, and breathing exercises involving controlled inflation of a balloon. These activities were intended as respiratory exercise components and should not be interpreted as MIP or MEP measurement procedures. The exercises were performed under supervision as part of the multicomponent training sessions.

All participants allocated to the intervention group completed the intervention period, and no intervention-related adverse events or protocol deviations were recorded. The total planned, including all participants, was a total of 180 planned sessions, of which 176 were completed, corresponding to an overall session adherence of 97.8%. No participants were withdrawn because of intervention-related reasons.

### 2.6. Statistical Analysis

Statistical analyses were performed using IBM SPSS Statistics for Windows (Version 21.0; IBM Corp., Armonk, NY, USA). The predefined primary outcome was diaphragm thickness. Secondary outcomes were functional walking capacity assessed using the 6-Minute Walk Test (6MWT) and health-related quality of life assessed using the Physical Health (PH) and Mental Health (MH) component scores of the SF-12. Hepatic attenuation was prespecified as an exploratory outcome. The distributional characteristics of the continuous variables were assessed using the Shapiro–Wilk test, together with inspection of model residuals and graphical assessment of the distributions. A two-way mixed-model analysis of variance (ANOVA) for repeated measures was performed to assess the effects of Group (intervention vs. control), Time (pre-intervention vs. post-intervention), and their interaction. The Group × Time interaction was the primary inferential test for determining whether the change from pre-intervention to post-intervention differed between the intervention and control groups. With only two assessment time points, the sphericity assumption was not applicable.

Before conducting the baseline-adjusted ANCOVA analyses, the homogeneity of regression slopes assumption was assessed separately for the SF-12 Physical Health (PH) and Mental Health (MH) components. For each outcome, an initial ANCOVA model included the group, the corresponding baseline score, and the group × baseline score interaction. A non-significant Group × baseline interaction was considered to indicate that the regression slopes were homogeneous across groups. Because this assumption was satisfied for both SF-12 PH and MH, the interaction terms were removed, and the final baseline-adjusted ANCOVA models were used to estimate adjusted between-group differences in post-intervention scores.

As a baseline-adjusted sensitivity analysis, analysis of covariance (ANCOVA) models was performed for each outcome using the corresponding baseline value as a covariate. These models were used to estimate the adjusted between-group difference in post-intervention outcomes while accounting for baseline values and were considered robustness analyses rather than additional primary confirmatory tests. Effect sizes for the mixed-model repeated-measures ANOVA were expressed as partial eta squared (*η*^2^p), with values of 0.01, 0.06, and 0.14 interpreted as small, medium, and large effects, respectively [25]. For the ANCOVA models, adjusted estimated marginal means and between-group mean differences were reported together with their corresponding 95% confidence intervals (95% CIs), F statistics, *p* values, and partial eta squared. For the prespecified primary outcome, diaphragm thickness, statistical significance was evaluated using a two-sided alpha level of 0.05. The secondary outcomes (6MWT, SF-12 PH, and SF-12 MH) and the exploratory hepatic attenuation outcome were not subjected to multiplicity adjustment.

Their *p*-values are therefore reported as nominal (unadjusted) values and are interpreted as hypothesis-generating rather than confirmatory evidence. Accordingly, the statistical interpretation throughout the manuscript gives primary emphasis to the prespecified primary outcome, while secondary and exploratory findings are interpreted more cautiously. In addition to *p*-values, between-group effect estimates and their corresponding 95% CIs were reported to facilitate interpretation of the magnitude and precision of the observed effects. Intra-observer reliability of the imaging measurements was assessed using the intraclass correlation coefficient (ICC). ICC values were interpreted as moderate (0.50–0.75), good (0.75–0.90), or excellent (>0.90) [26].

## 3. Results

**Figure 2:**
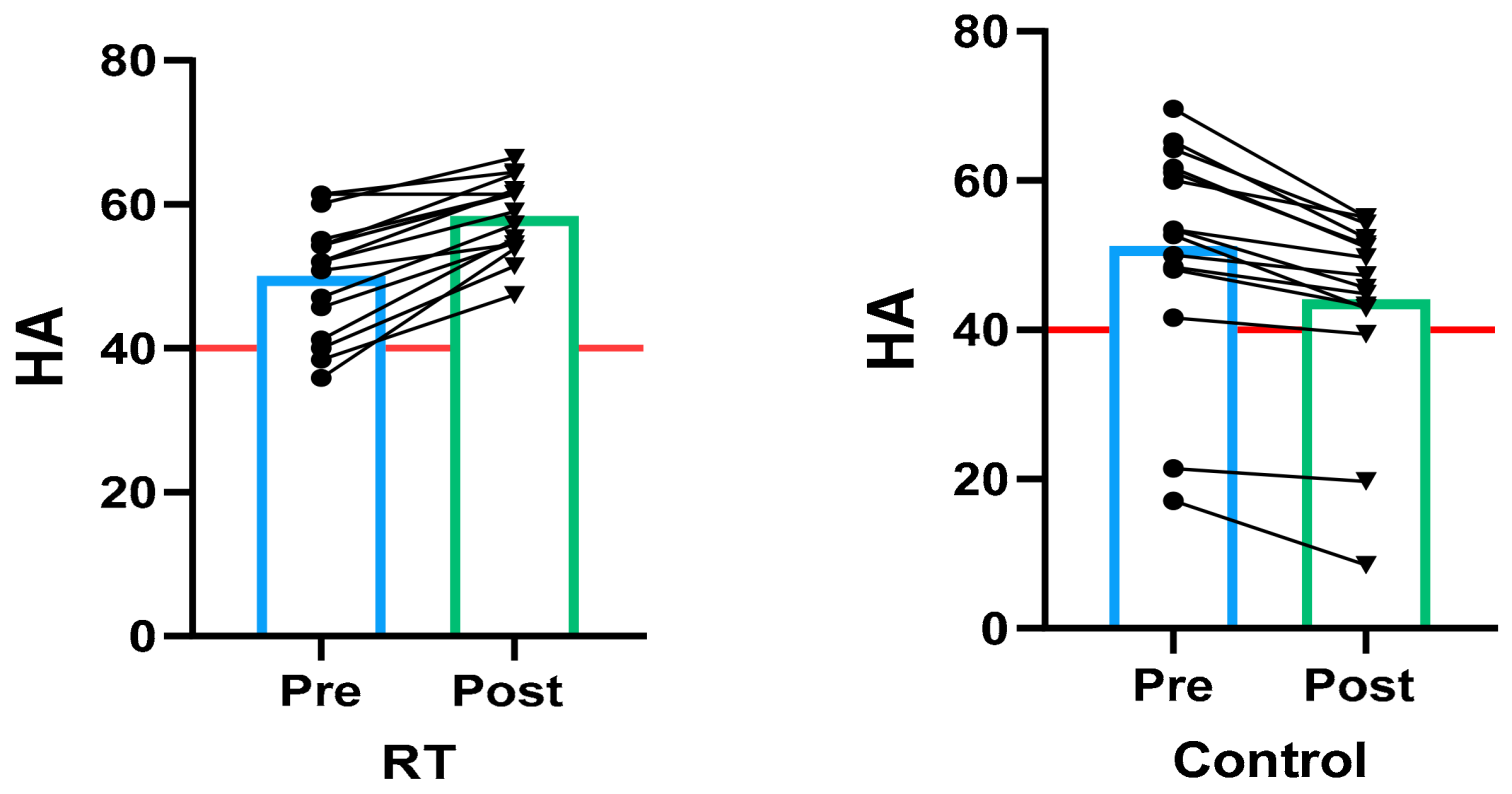
Comparison of pre- and post-training HA values.

**Figure 3:**
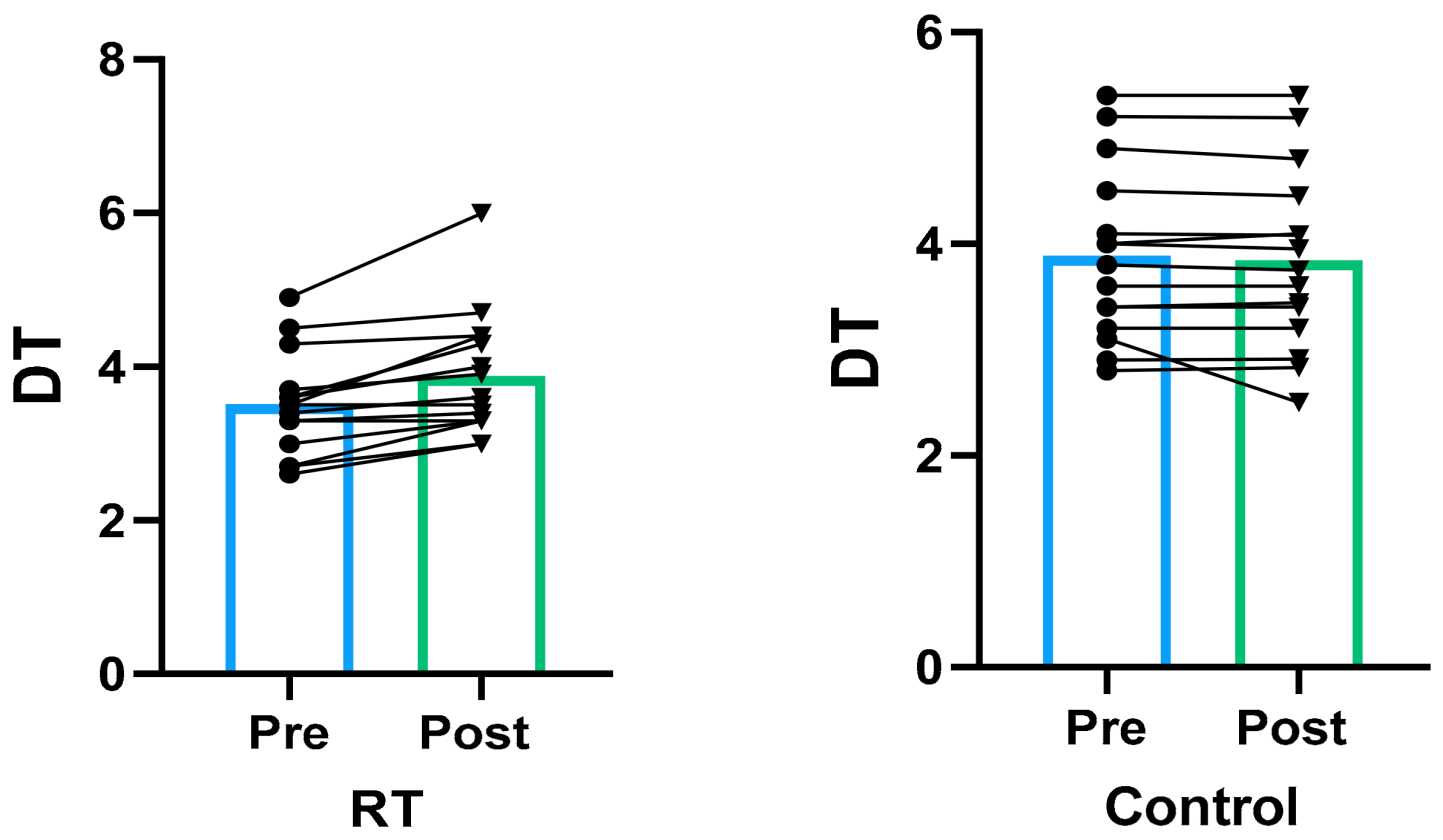
Comparison of pre- and post-training DT values.

**Figure 4:**
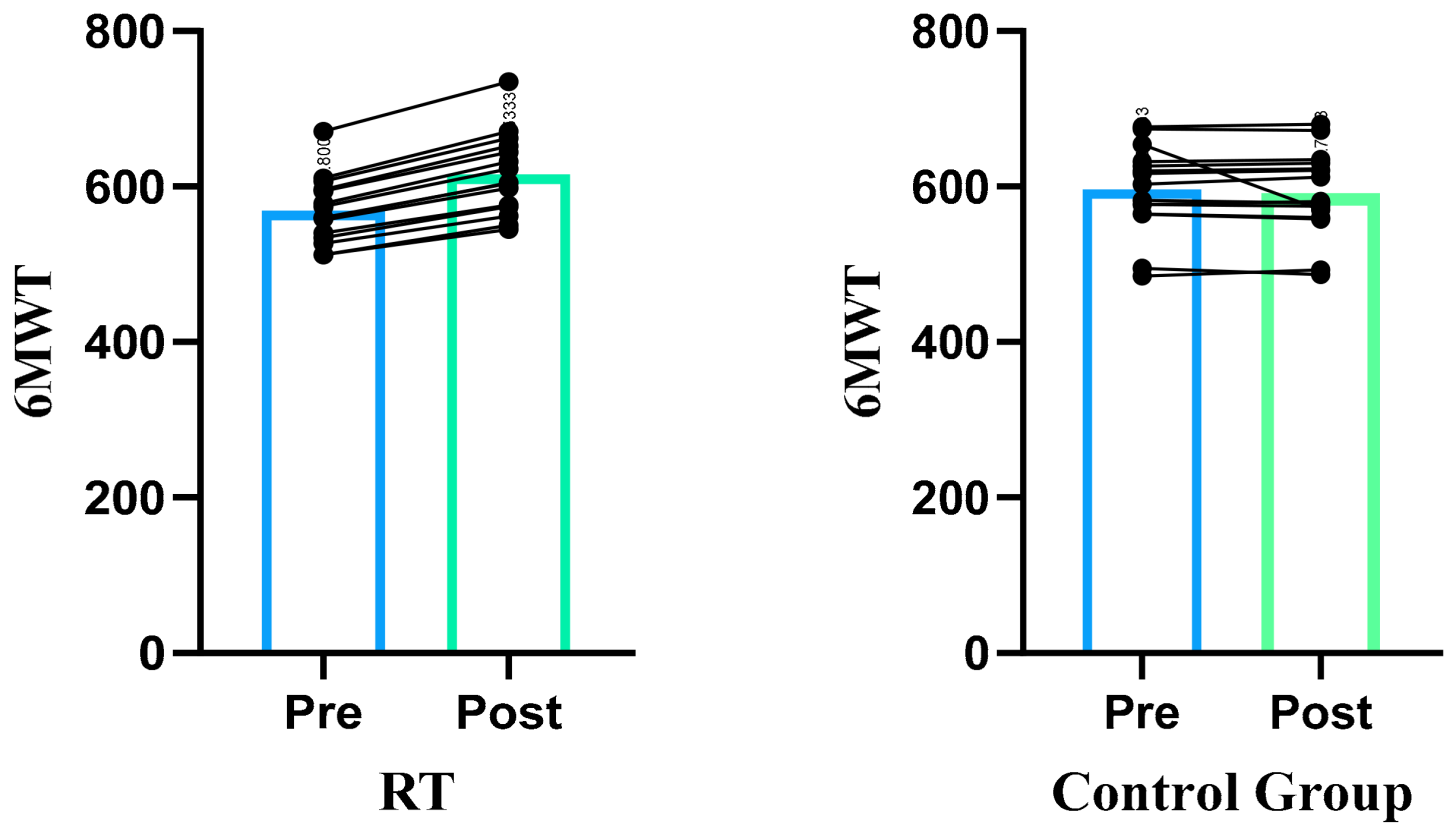
Comparison of pre- and post-training 6MWT values.

**Figure 5:**
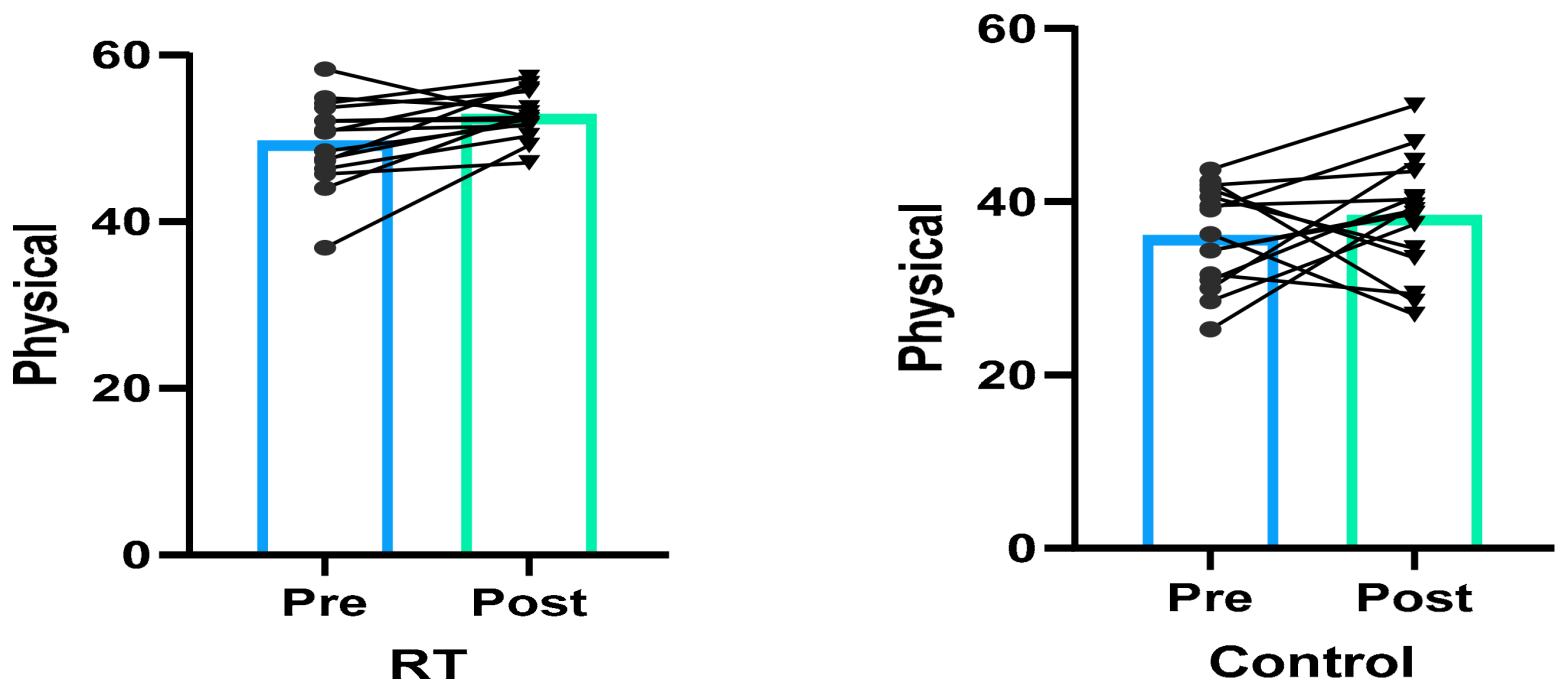
Comparison of pre- and post-training physical values.

Table 3, Table 4 and Table 5 and Figure 2, Figure 3, Figure 4 and Figure 5 show the changes between pre-test and post-test measurements in the resistance-based multicomponent training (RT) and control (CG) groups, as well as the time effect and group × time interactions. The findings indicate that the resistance-based multicomponent training had a strong and specific effect, particularly on diaphragm thickness, whereas the improvements observed in physical and mental health variables largely reflected time-dependent general effects. These findings suggest that the resistance-based multicomponent training was effective in increasing structural adaptations in the respiratory muscles but had a limited specific effect on perceived health dimensions.

Hepatic attenuation (HA), which was prespecified as an exploratory imaging outcome, increased from 49.98 ± 8.33 HU to 54.39 ± 7.11 HU in the RT group (8.82%) and decreased from 52.55 ± 9.25 HU to 49.51 ± 7.90 HU in the CG (5.78%). The main effect of Time was not statistically significant (F = 1.648, *p* = 0.210, *η*^2^p = 0.056), whereas the Group × Time interaction was statistically significant (F = 48.549, *p* < 0.001, *η*^2^p = 0.634). Because HA was prespecified as an exploratory outcome and was derived from a single-ROI non-contrast CT measurement, this finding is considered a nominal, hypothesis-generating association rather than confirmatory evidence of a change in hepatic fat content or hepatic steatosis.

A substantial augmentation in DT was detected in the RT following the resistance-based multicomponent training intervention. For the prespecified primary outcome, CT-derived diaphragm thickness increased from 3.51 ± 0.66 mm at baseline to 3.69 ± 0.63 mm after the intervention in the RT group, corresponding to an absolute increase of approximately 0.18 mm (5.12%). In the control group, diaphragm thickness changed from 3.45 ± 0.43 mm to 3.39 ± 0.48 mm. The Group × Time interaction was statistically significant (F = 21.058, *p* < 0.001, *η*^2^p = 0.429). In the baseline-adjusted sensitivity analysis, the adjusted post-intervention between-group difference in diaphragm thickness was 0.25 mm (95% CI, 0.14 to 0.36; F (1,27) = 20.902, *p* < 0.001, *η*^2^p = 0.436). These findings indicate a between-group difference in CT-derived diaphragm thickness following the intervention.

A substantial increase in 6MWT was detected in the RT following the resistance-based multicomponent training intervention. In the RT, the 6MWT value was 568.80 ± 42.87 m in the pre-test and increased to 615.53 ± 52.06 m in the post-test, corresponding to an 8.22% increase. Conversely, a marginal decline in 6MWT values was observed in the CG, amounting to a decrease of 0.85%. The time effect was found to be significant in repeated measurements (F = 44.101; *p* < 0.001; *η*^2^p = 0.612). Furthermore, the group × time interaction was also statistically significant (F = 66.604; *p* < 0.001; *η*^2^p = 0.704), indicating that the change in 6MWT reflected a group-specific intervention effect (Table 3, Figure 4).

SF-12 physical health (PH) scores increased over time in both groups. The PH score increased by 6.49% in the RT and by 6.38% in the CG. The group × time interaction was not significant (F = 0.135; *p* = 0.716; η^2^p = 0.005; Table 4, Figure 5), indicating no evidence of a differential change between groups over the study period.

**Figure 6:**
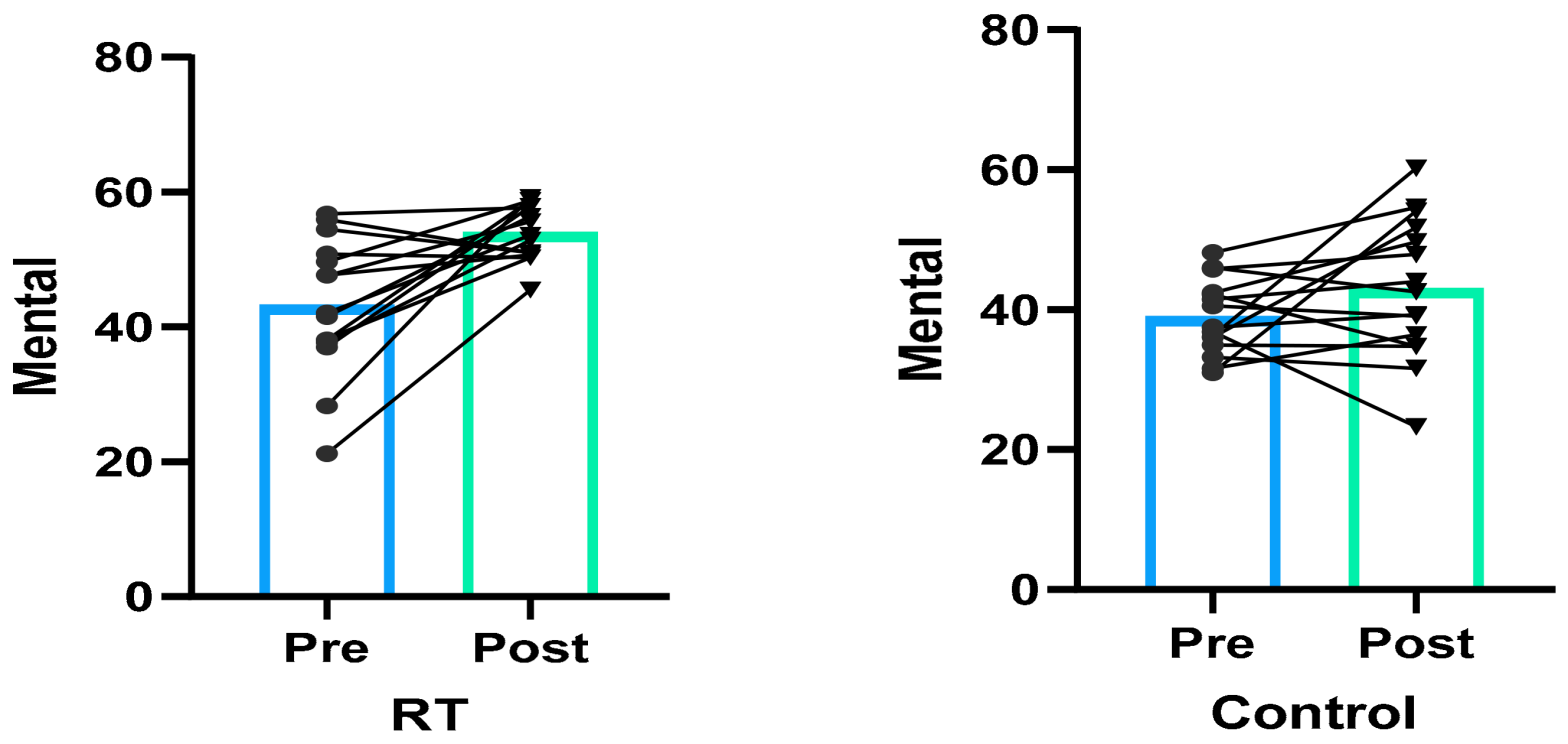
Comparison of pre- and post-training mental health values.

Similarly, an evaluation of mental health (MH) scores revealed a significant increase of 25.06% in the RT, with scores rising from 43.16 ± 10.15 to 53.98 ± 4.13. In contrast, the CG exhibited a more modest increase of 10.27%, with scores rising from 38.94 ± 5.36 to 42.94 ± 10.10. The group × time interaction did not attain the level of statistical significance (F = 3.183; *p* = 0.085; η^2^p = 0.102; Table 4, Figure 6).

In the baseline-adjusted ANCOVA sensitivity analysis, a statistically significant between-group difference in post-intervention SF-12 Physical Health scores was observed after adjustment for baseline values (*p* < 0.001). A statistically significant adjusted between-group difference was also observed for SF-12 Mental Health (*p* = 0.001). These baseline-adjusted findings were considered supplementary sensitivity analyses and were interpreted separately from the primary repeated-measures analysis. They were not interpreted as evidence of a statistically significant differential longitudinal change because the corresponding Group × Time interactions in the primary RM-ANOVA were not statistically significant.

To further evaluate the between-group treatment effects while accounting for potential baseline differences, baseline-adjusted ANCOVA models were performed for the primary and secondary outcomes. The corresponding adjusted estimated marginal means and between-group treatment effects with 95% confidence intervals are presented in Table 6. After adjustment for baseline values, the RT group demonstrated a significantly greater post-intervention diaphragmatic thickness than the control group. Similarly, significant between-group differences were observed for the 6-Minute Walk Test and both the physical and mental health components of the SF-12, favouring the RT group. For the exploratory hepatic attenuation outcome, the baseline-adjusted analysis also indicated a significant between-group difference, with higher post-intervention hepatic attenuation values in the RT group. The magnitude and precision of these between-group effects are presented as adjusted mean differences with corresponding 95% confidence intervals in Table 6.

## 4. Discussion

The present randomised controlled trial examined the short-term effects of a structured resistance-based exercise intervention on diaphragm morphology and broader health-related outcomes in healthy older men. The principal finding was a significant intervention-specific increase in diaphragm thickness after four weeks, with the resistance-based exercise group increasing from 3.51 ± 0.66 mm to 3.69 ± 0.63 mm, whereas a slight reduction was observed in the control group (group × time interaction: F = 21.058, *p* < 0.001, *η*^2^p = 0.429). Importantly, the intervention was also associated with a substantial improvement in functional walking capacity, with 6 min walk distance (6MWD) increasing by 46.73 m (8.22%) in the intervention group and showing a significant group × time interaction (F = 66.604, *p* < 0.001, *η*^2^p = 0.704). Together, these findings indicate that a relatively short exercise intervention was associated with both a measurable morphological response of the diaphragm and an objective improvement in functional walking capacity. However, the contribution of the individual resistance and respiratory components cannot be disentangled. However, these outcomes should be interpreted within their respective domains: the increase in diaphragm thickness represents a morphological response and does not by itself demonstrate improved respiratory muscle function, whereas the 6MWT provides evidence of improved walking performance. The absence of significant intervention-specific changes in SF-12 outcomes further suggests that short-term physiological and functional responses may occur without parallel changes in broader patient-reported health status. The hepatic attenuation finding should likewise be regarded as exploratory.

Importantly, the magnitude of the observed change in diaphragm thickness warrants cautious interpretation. The RT group demonstrated an absolute increase of approximately 0.18 mm (3.51 to 3.69 mm), corresponding to a 5.12% relative change. Although the statistical analysis demonstrated a significant between-group difference, statistical significance alone does not establish physiological or clinical relevance. The intra-observer reliability of the CT measurement was good (ICC = 0.823), indicating acceptable repeatability of the measurement procedure; however, ICC should not be interpreted as evidence that the observed change necessarily exceeds measurement error or represents a clinically meaningful threshold. Because a minimal clinically important difference or validated threshold for meaningful change in CT-derived diaphragm thickness in healthy older men has not been established in the context of this short-term intervention, the physiological and clinical significance of the approximately 0.18 mm increase remains uncertain. Accordingly, the present finding should be interpreted as a short-term morphological change in CT-derived diaphragm thickness rather than definitive evidence of respiratory muscle adaptation or improved respiratory function.

The increase in diaphragm thickness extends the concept of exercise-related skeletal muscle plasticity to the respiratory musculature. Previous studies have demonstrated that the diaphragm can adapt structurally and functionally to exercise-based interventions, although much of this evidence has been derived from inspiratory muscle training protocols or clinical populations [2,3,4,5,6,7,8,9,10,11,12,13,14,15,16,17,18,19,20,21,22]. The present findings provide preliminary evidence that a structured resistance-based exercise intervention may also be accompanied by measurable changes in diaphragm morphology in community-dwelling older adults. This observation is relevant to healthy aging because age-related alterations in respiratory muscle structure may contribute to reduced ventilatory reserve and exercise tolerance [2,3]. Nevertheless, the present result should be interpreted specifically as a change in diaphragm thickness rather than as evidence of improved diaphragm function. The CT protocol involved a single inspiratory-phase measurement and did not assess diaphragmatic excursion, respiratory muscle strength, contractile performance, thickening ratio, pulmonary function, or ventilatory efficiency. Accordingly, the observed increase should be considered an early morphological response rather than direct evidence of functional respiratory adaptation.

One possible explanation for the observed morphological response is that the mechanical and ventilatory demands associated with the exercise sessions may have increased respiratory muscle recruitment. Resistance exercise involves trunk stabilization, repeated bracing, and increased ventilatory demands, all of which may increase respiratory muscle activity. Previous research has demonstrated adaptive responses of the diaphragm to targeted respiratory exercise and changes in respiratory muscle recruitment and coordination following exercise-based interventions [2,3,4,5,6,7,8,9,10,11,12,13,14,15,16,17,18,19,20,21,22,23,24,25,26,27,28]. However, the present intervention also incorporated brief respiratory components, including pursed-lip/deep diaphragmatic breathing and MIP–MEP-related exercises, within the broader exercise program. Therefore, the observed increase in diaphragm thickness cannot be attributed exclusively to conventional resistance exercise. It is more appropriately interpreted as a response associated with the structured exercise intervention as a whole. Because electromyography, maximal inspiratory and expiratory pressures, diaphragm excursion, pulmonary function, and ventilatory efficiency were not assessed, proposed mechanisms such as increased neuromuscular recruitment, altered activation, or hypertrophic remodeling remain plausible but unconfirmed. In addition, the four-week duration does not permit determination of whether the observed morphological change represents true myofibrillar hypertrophy, transient structural adaptation, or a combination of structural and neuromuscular responses.

An important functional finding of the present study was the substantial improvement in six-minute walk distance (6MWD) following the four-week resistance-based exercise intervention. The intervention group increased from 568.80 ± 42.87 m at baseline to 615.53 ± 52.06 m after the intervention, corresponding to an absolute increase of 46.73 m (8.22%), whereas the control group demonstrated a slight reduction. The significant group × time interaction (F = 66.604, *p* < 0.001, *η*^2^p = 0.704) indicates that the improvement in walking capacity was substantially greater in the intervention group. This finding is broadly consistent with previous evidence suggesting that resistance exercise can improve functional walking performance in older men. In a systematic review and meta-analysis of healthy older adults, Smart et al. reported an approximately 31 m improvement in 6MWD following resistance exercise interventions of ≤24 weeks, although the magnitude of benefit varied across intervention characteristics and study populations [9]. Similarly, Ferté et al. reported improvements in 6MWT performance following resistance training in individuals with chronic obstructive pulmonary disease, with a mean improvement of approximately 37 m [21]. Recent intervention studies have also reported improvements in walking-related functional outcomes following short-term resistance exercise in older women and in postmenopausal women and breast cancer survivors, with changes of a similar order of magnitude to that observed in the present study [29,30,31]. These findings collectively suggest that functional walking capacity may respond measurably to relatively short periods of structured resistance-based exercise, although the magnitude and clinical relevance of the response may depend on participant characteristics, baseline functional status, intervention duration, and exercise modality.

Nevertheless, the present 6MWD finding should be interpreted within the heterogeneous evidence base. Not all studies have demonstrated a significant benefit of resistance-based multicomponent training on six-minute walking performance in older adults. Kashi et al. reported that, despite improvements in several domains of physical functioning and muscle strength, their systematic review and meta-analysis did not identify a significant overall benefit of resistance training for 6 min walk distance in adults aged ≥ 60 years [32]. Similarly, Maldonado-Martín et al. observed improvements in 6MWD in both exercise and attention-control groups among older adults with heart failure with preserved ejection fraction, highlighting the potential influence of temporal, familiarization, or nonspecific effects on repeated 6MWT performance [33]. Therefore, although the magnitude of the present between-group difference and the large group × time effect supports an intervention-associated improvement in functional walking capacity, the result should not be interpreted as evidence that resistance exercise consistently produces similar improvements across all older populations. Importantly, the concurrent increase in diaphragm thickness and 6MWD raises the possibility that morphological and functional adaptations may occur in parallel; however, the present study did not assess the association between these outcomes and therefore cannot establish a causal relationship. In particular, the improvement in 6MWD cannot be attributed specifically to the observed change in diaphragm thickness because the intervention included broader resistance-based and respiratory exercise components, while direct measures of respiratory muscle strength, diaphragm excursion, pulmonary function, and ventilatory efficiency were not obtained. The 6MWD should therefore be interpreted as an independent objective indicator of improved walking performance rather than as direct evidence of improved respiratory muscle function.

The health-related quality-of-life findings provide a more conservative perspective on the broader health effects of the intervention. SF-12 Physical Health scores increased by 6.49% in the intervention group and 6.38% in the control group, resulting in a significant time effect but no significant group × time interaction (*p* = 0.716). Mental Health scores also increased over time, with a numerically greater improvement in the intervention group, but the group × time interaction remained nonsignificant (*p* = 0.085). These findings indicate that the observed changes in perceived physical and mental health cannot be confidently attributed to the exercise intervention. Health-related quality of life is influenced by multiple interacting factors, including functional status, nutritional status, frailty, age, and subjective perceptions of health [10,11,12,13,34,35]. Although longer resistance-training interventions have demonstrated improvements in quality of life among older men [36], the relatively short four-week intervention and the healthy baseline characteristics of the present sample may have limited the likelihood of detecting an intervention-specific change. The divergence between the significant functional improvement in 6MWD and the absence of a significant SF-12 interaction is also informative, suggesting that objective functional responses may become detectable before broader patient-reported changes. This interpretation remains hypothesis-generating and requires confirmation through longer interventions using multidimensional health-related outcomes.

For the SF-12 outcomes, the primary repeated-measures analysis did not demonstrate statistically significant Group × Time interactions for either the Physical Health or Mental Health component. Although baseline-adjusted ANCOVA sensitivity analyses yielded statistically significant between-group differences in post-intervention scores, these findings should be interpreted cautiously because they represent adjusted cross-sectional between-group comparisons at post-intervention rather than evidence of differential longitudinal change. The discrepancy between the primary RM-ANOVA and baseline-adjusted ANCOVA results underscores the importance of interpreting the ANCOVA findings as supplementary rather than confirmatory evidence. The homogeneity of regression slopes assumption was assessed separately for the two SF-12 components. For SF-12 Physical Health, the Group × baseline PH interaction was not statistically significant (F (= 0.268, *p* = 0.609). Similarly, the Group × baseline MH interaction was not statistically significant for SF-12 Mental Health (F = 0.706, *p* = 0.408). Thus, the homogeneity of regression slopes assumption was satisfied for both outcomes, supporting the subsequent baseline-adjusted ANCOVA models.

The hepatic attenuation findings require the greatest degree of caution. A significant group × time interaction was observed, with hepatic attenuation increasing in the intervention group and decreasing in the control group. However, hepatic attenuation was an exploratory outcome and was assessed using a single region of interest on non-contrast CT. This approach does not provide an equivalent assessment to validated quantitative techniques such as MRI-proton density fat fraction or controlled attenuation parameter and therefore cannot establish changes in hepatic attenuation or diagnose hepatic steatosis. The direction of the between-group change is nevertheless of interest because previous research suggests that exercise can influence hepatic fat and metabolic pathways [8,37,38]. However, the present study did not assess liver enzymes, insulin sensitivity, dietary intake, body composition, or other metabolic mediators that could explain changes in hepatic attenuation. Therefore, this finding should be considered hypothesis-generating rather than confirmatory, and future studies should use validated quantitative imaging together with direct metabolic assessments.

From a healthcare and healthy-ageing perspective, the combined findings are potentially relevant because the intervention was associated with improvements in both an objective measure of functional walking capacity and a morphological measure of the respiratory musculature. Resistance-based exercise is already an established component of preventive exercise strategies for older adults because of its effects on strength, physical performance, and maintenance of functional capacity [2,4,6]. The present findings broaden this perspective by suggesting that structured exercise may also be associated with measurable changes in respiratory muscle morphology. The 6MWD response is particularly relevant to the Special Issue’s emphasis on functional outcomes because walking capacity is an accessible indicator of mobility and physical performance in older men. An important consideration is that the intervention should not be interpreted as resistance training in isolation. The four-week programme was multicomponent and incorporated conventional resistance exercises together with diaphragmatic breathing, pursed-lip breathing, balloon-inflation breathing exercises, mobility, balance activities, and a dynamic warm-up. Therefore, the observed increase in diaphragm thickness cannot be attributed specifically to resistance exercise. The respiratory components represent a biologically plausible contributor to the observed diaphragm response, whereas the contribution of the individual program components cannot be disentangled within the present study design. Future studies using factorial or component-specific designs are warranted to determine the independent and combined effects of resistance and respiratory exercise on diaphragm morphology. Similarly, the improvement in 6MWD should not be interpreted as evidence of improved respiratory function. From a preventive-healthcare perspective, the findings instead support the feasibility of structured exercise as a multidimensional strategy for preserving functional and physiological capacity during ageing.

### 4.1. Limitations

Several strengths and limitations should be considered when interpreting these findings. The randomised controlled design, supervised intervention, homogeneous sample, blinded outcome assessment, and predefined primary outcome strengthen the internal consistency of the study. Diaphragm and hepatic attenuation measurements also demonstrated good intra-observer reliability. However, the sample was restricted to healthy community-dwelling older men, limiting generalisability to women, frail individuals, individuals with chronic disease, and other age groups. The four-week intervention also limits conclusions regarding longer-term structural, functional, and health-related adaptations. Diaphragm thickness was derived from a single-slice CT measurement rather than ultrasound or MRI and therefore cannot characterise diaphragm excursion, contractile behaviour, or regional morphological adaptations. The hepatic imaging outcome should also be interpreted cautiously. Hepatic attenuation was assessed using a single region of interest on non-contrast CT and was included as an exploratory imaging measure. Although changes in hepatic attenuation may be related to differences in hepatic fat accumulation, the present approach does not directly quantify hepatic fat content and is not equivalent to validated quantitative methods such as MRI-PDFF or controlled attenuation parameter measurements. Therefore, the observed change in hepatic attenuation should not be interpreted as definitive evidence of a change in hepatic fat content or fatty liver disease. Future studies should incorporate validated quantitative techniques, preferably MRI-PDFF or other established methods, to directly quantify hepatic steatosis. Dietary intake, habitual physical activity, and hydration status were not objectively monitored, limiting attribution of secondary and exploratory changes exclusively to the intervention. An important limitation is that the intervention was multicomponent and included both resistance and respiratory exercises. Therefore, the present design does not allow for the independent contribution of conventional resistance exercise and respiratory exercise components to the observed changes in diaphragm thickness to be determined. Accordingly, the observed diaphragmatic response should be interpreted as an effect of the overall resistance-based multicomponent training programme rather than as an isolated effect of resistance exercise. Future studies using factorial or component-specific designs are warranted to determine the relative contribution of resistance and respiratory training to diaphragmatic adaptations. Another limitation is that diaphragm assessment was restricted to CT-derived diaphragm thickness. Although the measurement demonstrated good intra-observer reliability (ICC = 0.823), no formal measurement-error analysis or minimal clinically important difference was established for the observed change, and therefore the clinical relevance of the approximately 0.18 mm increase cannot be determined. Furthermore, diaphragm excursion, thickening fraction, maximal inspiratory and expiratory pressures, spirometry, electromyography, and ventilatory-efficiency measures were not obtained. Consequently, the observed change in diaphragm thickness should not be interpreted as evidence of improved respiratory muscle function or broader respiratory physiological adaptation. Finally, although the 6MWT provided an objective measure of functional walking capacity, it was the principal functional performance measure and was not accompanied by complementary assessments such as gait speed, lower-limb strength, chair-stand performance, or cardiopulmonary exercise testing. Future studies should therefore incorporate larger and more heterogeneous samples, longer interventions, validated respiratory measures, and a broader battery of functional and metabolic outcomes.

### 4.2. Practical Implications

From a healthcare perspective, the principal implication of this study is not that resistance exercise improves respiratory function per se, but that a feasible structured exercise intervention may be associated with measurable changes in respiratory muscle morphology and functional walking capacity during aging. The significant improvement in 6MWD suggests that relatively short-term supervised exercise may provide an accessible approach for supporting functional mobility in healthy older men. This is consistent with the broader role of resistance exercise in preventive exercise strategies targeting strength, physical performance, and functional independence [1,3,4,5,6]. However, the findings should be interpreted as supporting exercise-based healthy-aging strategies rather than as evidence for a specific diaphragm-directed therapeutic effect. Exercise prescription should remain individualized according to health status, baseline functional capacity, exercise history, and safety considerations.

### 4.3. Future Research

Future research should determine whether the morphological diaphragm response observed after short-term exercise translates into measurable improvements in respiratory muscle function. Larger randomized trials with longer follow-up should combine diaphragm imaging with maximal inspiratory and expiratory pressures, ultrasound-based excursion and thickening measures, pulmonary function, and cardiopulmonary exercise testing. Because the present intervention included both resistance and respiratory exercise components, factorial or adequately controlled designs would also be valuable for determining the independent and combined effects of resistance exercise and inspiratory muscle training. Further studies should examine whether changes in diaphragm morphology are associated with changes in 6MWD, rather than assuming a causal relationship between these outcomes. In addition, broader functional assessments, including gait speed, chair-stand performance, strength, balance, and mobility measures, would clarify whether the observed improvement in 6MWD represents a wider improvement in functional capacity. Finally, longer interventions incorporating validated hepatic imaging, metabolic biomarkers, dietary monitoring, and patient-reported outcomes may clarify whether the exploratory hepatic finding and the functional responses observed in the present study persist over time.

## 5. Conclusions

A short-term resistance-based multicomponent training programme incorporating respiratory exercises may increase diaphragm thickness and improve functional capacity in healthy older men. Given the multicomponent nature of the intervention, these findings should not be interpreted as evidence of an isolated effect of conventional resistance exercise on diaphragmatic morphology. The concurrent increase in 6 min walk distance suggests that short-term exercise may produce measurable functional benefits during ageing, although the observed change in diaphragm thickness should be interpreted as a morphological response rather than direct evidence of improved respiratory muscle function. No significant intervention-specific changes were observed in health-related quality of life, whereas the hepatic attenuation finding should be considered exploratory because of the limitations of single-ROI non-contrast CT assessment. Overall, these findings support further investigation of structured exercise as a component of exercise-based healthy ageing strategies, particularly through longer and adequately powered trials incorporating validated respiratory, functional, and metabolic outcomes.

## Figures and Tables

**Table 1:** Descriptives.

Descriptives	Control (n:15)		RT (n:15)	
	X	S. D	X	X
Age	65.27	4.65	68.27	3.81
Height	177.07	4.95	176.93	3.45
Weight	83.53	3.85	82.93	3.73
BMI	26.65	0.84	26.48	0.47

**Table 2:** Resistance-based Multicomponent Training Program and Protocols.

	Exercise	Sets × Reps	Description
Day 1 Monday	Chair Sit-to-Stand (Squat)	3 × 12	At knee level, slow and controlled
	Glute Bridge	3 × 15	Lying on the floor or bed
	Standing Hip Abduction (with Resistance Band)	2 × 12/leg	Balance support may be required
	Core: Seated Knee Raises	2 × 10	While seated, pull one knee toward the chest
	Respiratory: Pursed-Lip Breathing + Deep Diaphragmatic Breathing	2 × 1 min	Inhale through the nose, exhale slowly through pursed lips
Day 2 Wednesday	Wall Push-Up	3 × 10–12	Body straight, hands at shoulder height
	Seated Row with Resistance Band	3 × 12	The band can be fixed to the chair
	Lateral Shoulder Raise (with Dumbbell or Water Bottle)	2 × 12	Lift arms to shoulder height
	Biceps Curl	2 × 12	With light dumbbells
	Respiratory: structured inspiratory and expiratory breathing exercises involving balloon inflation	3 × 5 breaths	Using balloon inflation
Day 3 (Friday)	Step-Up/Down on Stairs	3 × 6–10 steps	Slow and careful, use support if needed
	Mini Squat + Ball Chest Press	2 × 10	Controlled chest press using a ball against the wall
	Single-Leg Balance (Standing)	2 × 20 s/leg	Use wall support if necessary
	Hip Stretch + Trunk Rotation	2 × 8	Slow and rhythmic movement
	Respiratory: Diaphragmatic Breathing + Trunk Movement	2 × 1 min	Hands on abdomen, seated or standing

**Table 3:** Comparison of pre- and post-training DT, HA and 6MWT values.

Outcome	Group	Pre-Test	95 CI		Post-Test	95 CI		Δ%	Time Effect		Group × Time	
		X̅ ± SD	L.B.	U.B.	X̅ ± SD	L.B.	U.B.		F	p(*η*^2^p)	F	p(*η*^2^p)
DT	RT	3.51 ± 0.66	3.21	3.8	3.69 ± 0.63 ↑	3.4	3.99	5.12	5.824	0.023 (0.172)	21.058	<0.001 (0.429)
	CG	3.45 ± 0.43	3.15	3.74	3.39 ± 0.48 ↓	3.09	3.68	−1.28				
HA	RT	49.98 ± 8.33	45.33	54.63	54.39 ± 7.11 ↑	50.42	58.37	8.82	1.648	0.210 (0.056)	48.549	<0.001 (0.634)
	CG	52.55 ± 9.25	47.89	57.2	49.51 ± 7.9 ↓	45.33	53.48	−5.78				
6MWT	RT	568.80 ± 42.87	542.28	595.32	615.53 ± 52.06	586.97	644.10	8.22	44.101	<0.001 0.612	66.604	<0.001 0.704
	CG	596.8 ± 56.5	570.01	623.06	591.73 ± 55.87	563.17	620.3	−0.85				

DT, Diaphragm thickness; HA, Hepatic attenuation; Δ%, Percentage change rate, ↑ increased, ↓ decreased.

**Table 4:** Comparison of pre- and post-training SF-12 values.

Outcome	Group	Pre-Test	95 CI		Post-Test	95 CI		Δ%	Time Effect		Group × Time	
		X̅ ± SD	L.B.	U.B.	X̅ ± SD	L.B.	U.B.		F	p(*η*^2^p)	F	p(*η*^2^p)
PH	RT	49.56 + 5.26	46.64	52.47	52.78 + 2.79↑	50.01	55.55	6.49	4.760	<0.038 (0.145)	0.135	0.716 (0.005)
	CG	36.01 + 5.76	33.09	38.93	38.31 + 6.86↑	35.54	41.08	6.38				
MH	RT	43.16 + 10.15	38.87	47.45	53.98 + 4.13↑	49.9	58.06	25.06	14.999	<0.001 (0.349)	3.183	0.085 (0.102)
	CG	38.94 + 5.36	34.65	43.24	42.94 + 10.10↑	38.86	43.24	10.27				

PH, Physical health; MH, Mental health; Δ%, Percentage change rate.

**Table 5:** Assessment of the homogeneity of regression slopes for SF-12 outcomes.

Outcome	Interaction Term	F	df1	df2	*p*-Value	Interpretation
SF-12 Physical Health	Group × Baseline PH	0.268	1	26	0.609	Assumption satisfied
SF-12 Mental Health	Group × Baseline MH	0.706	1	26	0.408	Assumption satisfied

Note: The homogeneity of regression slopes assumption was assessed separately for each SF-12 component by testing the Group × baseline outcome interaction in an ANCOVA model including the corresponding baseline score, group, and their interaction. Non-significant Group × baseline interactions were interpreted as supporting the assumption of homogeneous regression slopes. Because the assumption was satisfied for both SF-12 components, the interaction terms were removed and the final baseline-adjusted ANCOVA models were used.

**Table 6:** Baseline-adjusted ANCOVA results and estimated between-group treatment effects.

Outcome	CG Adjusted Mean (95% CI)	RT Adjusted Mean (95% CI)	RT − CG Adjusted Difference (95% CI)	Baseline-Adjusted Group Effect F (1,27)	*p*	*η*^2^p
DT (mm)	3.42 (3.34, 3.50)	3.66 (3.59, 3.74)	0.25 (0.14, 0.36)	20.902	<0.001	0.436
HA	48.47 (47.17, 49.77)	55.43 (54.13, 56.73)	6.96 (5.11, 8.82)	59.464	<0.001	0.688
6MWT (m)	577.58 (568.08, 587.09)	629.68 (620.18, 639.18)	52.10 (38.40, 65.80)	60.882	<0.001	0.693
PH	39.40 (35.65, 43.14)	51.69 (47.95, 55.44)	12.30 (5.93, 18.66)	15.717	<0.001	0.368
MH	43.19 (38.99, 47.39)	53.73 (49.53, 57.93)	10.54 (4.49, 16.59)	12.786	0.001	0.321

Note. ANCOVA models were adjusted for the corresponding baseline value of each outcome. Adjusted means represent estimated marginal means at the post-intervention assessment. Between-group treatment effects are presented as the adjusted mean difference between the intervention and control groups, with corresponding 95% confidence intervals (95% CIs). Positive values indicate higher post-intervention values in the intervention group. F statistics represent the adjusted between-group effect. *η*^2^p, partial eta squared; CI, confidence interval; ANCOVA, analysis of covariance.

## Data Availability

The data used in this study can be accessed at https://doi.org/10.6084/m9.figshare.31536367.

## Abbreviations

The following abbreviations are used in this manuscript:

RT	Resistance-based Multicomponent Training Group
CG	Control Group
ANOVA	Analysis of Variance
RCT	Randomized Controlled Trial
ADL	Activities of Daily Living
ICC	Intraclass Correlation Coefficient
ROI	Regions of İnterest
CAP	Controlled Attenuation Parameter
HU	Hounsfield Units
RPE	Perceived Exertion
NSCA	National Strength and Conditioning Association
DT	Diaphragm Thickness
MH	Mental Health
